# The burning issue of white phosphorus: a case report and review of the literature

**DOI:** 10.1186/s40696-017-0034-y

**Published:** 2017-08-30

**Authors:** Uri Aviv, Rachel Kornhaber, Moti Harats, Josef Haik

**Affiliations:** 1 Department of Plastic and Reconstructive Surgery, Sheba Medical Center, Tel Hashomer, Israel; 2 Sackler School of Medicine, Tel Aviv University, Tel Aviv, Israel; 3 School of Health Sciences, University of Tasmania, Rozelle, NSW Australia; 4 Institute for Health Research, University of Notre Dame, Fremantle, Australia; 5 Talpiot Leadership Program, Sheba Medical Center, Tel Hashomer, Israel

**Keywords:** Chemical burn, White phosphorus, Full thickness, Treatment outcome, Wound healing, Case report

## Abstract

**Background:**

Burns from white phosphorus are rare and remain a challenge for clinicians. White phosphorus burns are often associated with smaller surface areas and high morbidity rates. Classed as a chemical burn, white phosphorus is used for military purposes and within industry, for the manufacture of fireworks and agricultural products.

**Case presentation:**

In this report, we discuss the case of a 40 years old female who sustained 2% Total Body Surface Area partial to full thickness burns from white phosphorus. The burns were treated conservatively with mafenide acetate on the medial calf and dorsum of foot and Flaminal Forte was used for the palmar region. The patient was discharged 22 days after admission and followed up in the outpatient clinic. Despite the use of pressure garments, hypertrophic scarring began to develop on the dorsum of her right foot.

**Conclusions:**

During peacetime, white phosphorus possess a significant danger to civilians. Awareness of the unique nature of white phosphorus among military burn clinicians should be emphasized.

## Background

In 1669, a German merchant and alchemist, Hennig Brand (1630–1692) who was intent on searching for the “Philosopher’s stone”, a legendary object that could turn base metals into gold, was unintentionally preparing white phosphorus [1, 2]. Brand prepared a mixture of dried urine and sand by means of boiling [1, 2]. As a result, the vapors created were passed through water causing the white  phosphorus to become a soft, waxy, white solid material [1, 2]. Hence the new element was named, phosphorus deriving from the Greek word *phosphoros* (Φωσφόρος) meaning (light bringer or bearer) [1].

With a melting point of 44 °C, white phosphorus is insoluble in water [3] and above 30 °C, the particles spontaneously oxidize when in contact with the atmosphere forming phosphorus pentoxide [4] a strong desiccant and dehydrating agent. Due to its dehydrating action, white phosphorus is a highly corrosive substance [3]. In the dark, white phosphorus emits a greenish light and white fumes can be seen and a garlic-like odor [5]. The bulk of phosphorus produced with this method is then converted to phosphoric acid that is used in agriculture for the production of fertilizers [1, 2] rodenticides, fireworks and doping agent for silicon in the manufacturing of semiconductors [6].

Well known for its use in military warfare, white phosphorus burns displaying an illuminous yellow flame producing a thick white smoke that serves as a smokescreen, interferes with infrared cameras and weapon tracking devices [6]. For military personnel, materials as white  phosphorus present a high risk for burns from incendiary shells and detonators [7] and such injuries warrant the expertise of experienced military burn surgeons.

Here we present the case of a 40-year-old female who sustained chemical burns from contact with white phosphorus.

## Case presentation

We report a case of a white  phosphorus burn that has a unique mechanism of injury involving a 40-year-old woman with her 7-year-old daughter who were collecting rocks on a beach in Tel Aviv. Upon returning home, the daughter washed the rocks of sand with tap water and immediately wrapped them in a paper towel. According to her anamnesis, the daughter presented her mother an unusual appearing, yellow translucent colored rock covered by a wet paper towel. As she commenced unwrapping the paper towel, white smoke emanated from the rock that was followed by ignition of flames and accompanied by a severe burning sensation in her hands. Subsequently, she dropped the rock which made contact with her right calf and then landed on her right foot that was covered by a sock and ignited. The patient then proceeded to put out the flames in the bathroom shower. Upon return to the living room, the piece of white  phosphorus had ignited the couch which took hold and the entire apartment was gutted with fire. Both mother and child were safely evacuated from the apartment with no further injuries sustained.

Upon arrival at the Emergency Department, within 1 h of the burn injury, the patient presented as conscious and talking with no evidence of an inhalation injury. Assessment revealed chemical burns from contact with what was suspected to be white  phosphorus. No adequate first aid was administered at the time of the injury. First aid was commenced and the burns underwent decontamination, irrigation and debridement of devitalised tissue. Her wounds were thoroughly irrigated with water and then covered with saline soaked pads and was subsequently admitted to our Burns Center for further treatment.

Digital photography with written informed consent from the patient pertaining to the use for treatment and teaching and learning purposes were taken on admission to the Emergency Department [8]. As this was a retrospective case report the ethics committee of Sheba Medical Center Helsinki Committee does not require ethics approval to be sought for this case report.

**Fig. 1:**
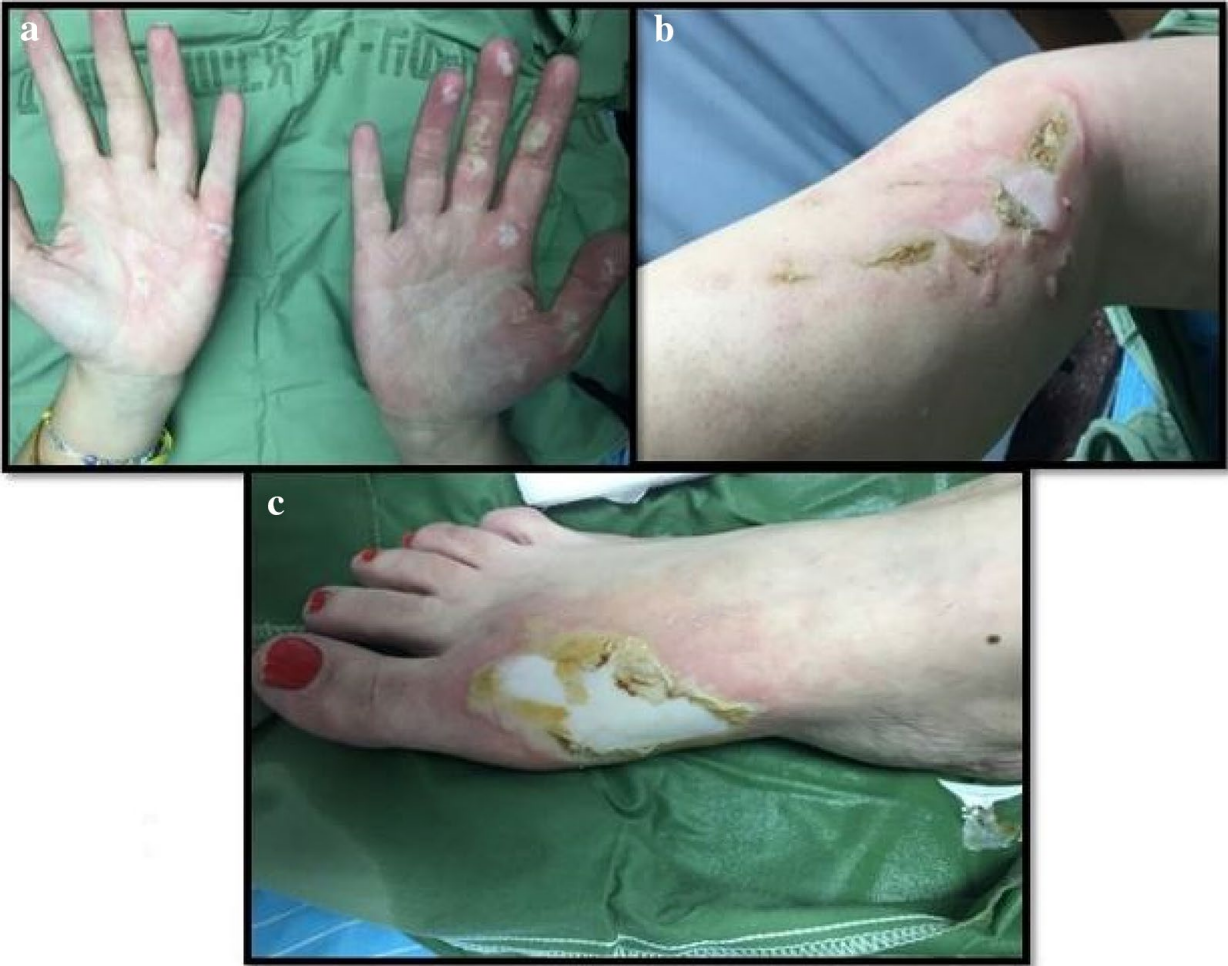
White phosphorus burns on presentation to the Emergency Department; **a** Partial thickness burns sustained to palmer surface of bilateral hands; **b** Deep dermal to full thickness burns sustained to right medial calf; **c** Full thickness burns sustained to dorsum of right foot

Scattered partial thickness burns were sustained on bilateral hands to the dorsum and palms aspects of 0.5% Total Body Surface Area (TBSA), deep dermal burns to her right medial calf of 1% TBSA and full thickness burns were sustained to her right foot dorsum aspect calculated to be 0.5% TBSA (see Fig. 1). Due to the small surface area involvement and our Department’s experience with conservative non-surgical approach for minor burn care, the patient’s burns were treated with mafenide acetate (Sulfamylon) on the right medial calf and dorsum of foot and Flaminal Forte for the palmar wounds. Vital signs were all within normal limits, bloods were unremarkable and electrocardiogram abnormalities were not observed.

**Fig. 2:**
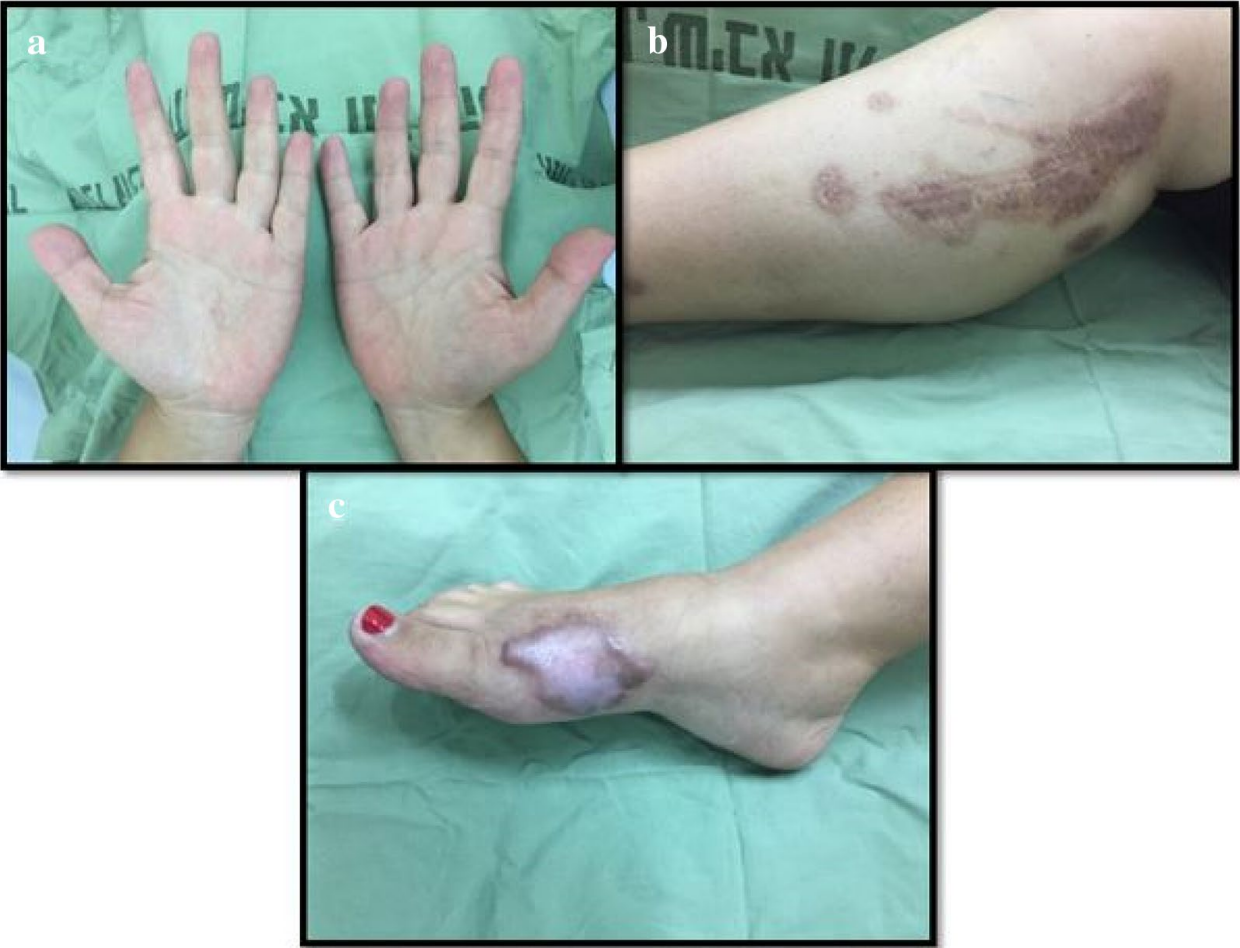
Plates **a, b** and **c** 7 months post burn injury from white phosphorus. **b** Scarring seen on right medial calf **c** hypertrophic scarring evident on right foot

Throughout the hospitalization period, blood tests were routinely taken twice weekly. The phosphorus level increased gradually from 2.90 mg/dL on day 1 to 4.40 mg/dL on day 14 (normal range 2.5–4.5 mg/dL). C-reactive protein (CRP) levels increased throughout the first 5 days and reached 122.21 mg/L, then gradually decreased to normal parameters (normal range 0–5 mg/L). During the admission period, occupational therapy and physiotherapy were provided to facilitate full range of motion to the hands and right leg. The patient was discharged 22 days after admission and followed up in the outpatient clinic. Pressure garments were provided however; hypertrophic scars began to develop on the dorsum of her right foot. Silicone sheets were provided under the pressure garment and the patient was educated on the importance of adherence to burn care therapy. The patient was again followed up 7 months post injury where digital images were once again obtained with the consent of the patient (see Fig. 2).

Due to the unusual appearance of the burn in this case report, the patient’s clothes were sent for examination to a forensic specialist. Consequently, the police and military investigators confirmed the nature of the chemical agent as white phosphorus crystals to permit preventive measures and announcements were made in the media. It is not known exactly how the piece of white  phosphorus came to be on the beach in Tel Aviv however, the most probable explanations could be that the it may have fallen of a passing cargo ship, migrated from distant industrial factories or power station, remnants from naval training exercises or even an old incendiary bombshell that has been carried away by the sea currents.

## Discussion

The aim of this case review was to highlight white phosphorus as a mechanism of injury for deep chemical burns and the limited literature available to guide current practice. Burns sustained from white phosphorus are relatively rare and are reported to be of a small surface area, deep and accompanied with significant life threatening physiological effects [4, 9] also highlighted in Saracoglu et al.’s [10] case report. Furthermore, white phosphorus burns sustained during military combat are noted to have a greater TBSA as identified by Karunadasa et al.’s [11] case report.

Reports of white phosphorus cutaneous burns within the peer reviewed literature began to emerge in the 1940s [12, 13]. Both Jones [13] and Godding and Notton [12] discussed the treatment of white phosphorus burns that both heavily feature the use of copper sulfate. The use of copper sulfate to treat  phosphorus burns dates back over 100 years to mainly destroy minute  phosphorus particles and also to blacked the large  phosphorus particles for removal [14].

Highly fat soluble, white phosphorus absorption results in necrosis of the liver or kidney [4, 14]. Although not evident in our case, white phosphorus can produce serious physiological alterations including hypocalcemia, hyperphosphatemia with calcium-phosphate shifts, as soon as 1 h after the burn is sustained [4, 15]. The rapid development of hypocalcemia and hyperphosphatemia is responsible for cardiac arrhythmias with abnormalities post burn including prolonged QT intervals, ST-T wave changes and progressive bradycardia [4, 5, 9]. Furthermore, a relatively small surface area of 10–15% TBSA can evoke a sudden and often unexpected death [16]. Excision of the area within 1 h of the burn is reported not to improve survival rates [5]. This therefore suggests that metabolic changes due to exposure to white phosphorus occur early [5]. As seen in Fig. 1, a greyish discoloration is produced almost immediately when white phosphorus comes in contact with the skin [17]. Burns from white phosphorus are reported be intensely painful and are akin to those from hydrofluoric acid which are reported to be more severe than burns caused from caustic soda and sulphuric acid [17].

Treatment modalities for white phosphorus have historically been controversial and an area of debate due to its toxic effect. As highlighted by Barillo et al. [4] and Summerlin et al. [14], the use of copper sulfate in the treatment of white phosphorus has evolved over the past century primarily for the identification of phosphorus particles. When in contact with copper sulfate, the phosphorus turns black for easy identification and subsequent removal [14]. However, copper sulfate is absorbed through the burnt skin and has been reported to have adverse effects including vomiting, diarrhea, oliguria, hematuria, hemolysis, hepatic necrosis, tachycardia and hypotension [5, 14]. Within the burns literature, Summerlin et al. [14] reported three cases of copper toxicity within 15 h of using 2% copper sulfate solution. Therefore, it is surprising that others reported the use of copper sulfate solution within the peer reviewed literature. Barillo et al. [4] strongly advocates that the use of copper sulfate in the treatment of white phosphorus burns has no place in contemporary burn management. Subsequently, the use of a Wood’s lamp facilitates the identification of residual luminescent phosphorus particles that poses minimal adverse effects with particles appearing fluorescent under the ultraviolet light [18]. This is also raised by Saracoglu et al. [10] as copper sulfate is potentially systemically toxic. Other options that have been reported include 1–3% solution of silver nitrate as a safer option to copper sulfate [4].

As white phosphorus become liquid at 44 °C, it is critical that the use of warm water is avoided and as reported in our case, saline soaked gauze/pads were used to cover the wound. Subsequently, Karunadasa et al. [11] advocates the use of saline soaked gauze for wound coverage that facilitates oxygen depletion to any remaining phosphorus particles. The primary goal is to stop the burning process and immediately remove any contaminated clothing and shoes. Irrigation with cool, as opposed to warm, water is necessary [4, 9]. However, aggressive irrigation with copious amounts of water may result in adverse effects with phosphorus particles transported to uninvolved areas of the skin and reignite when exposed to the air.

Our case report resonates with Frank et al. [9] who also reports exceptional circumstances in which white phosphorus rocks found whilst strolling on a beach in Germany were mistaken for amber which subsequently spontaneously ignited in peoples’ pockets sustaining small deep burns. For burn clinicians, great care needs to be exercised when removing the burning agent, as highlighted by Barqouni et al. [19] who reported in their case report that during debridement of white phosphorus burns, a particle of white phosphorus became dislodged resulting in a nurse sustaining a superficial burn to the neck. This further highlights the need for meticulous occupational health and safety that is required when treating white phosphorus burns.

There remains a paucity of literature concerning best practice for the management of white phosphorus burns. A recent comprehensive Cochrane systematic review of interventions for treating phosphorus burns [20] highlights this very point containing only two retrospective studies emanating from the 1960s and 1970. Given the advancements in burn care and science over the past 50 years, an integration of the available peer reviewed literature is necessary to guide best practice. Calls for higher-level evidence is required however, due to the rare incidence, this remains methodologically challenging.

## Conclusion

White phosphorus burns have a unique nature making both diagnosis and management complex and challenging for clinicians. Considering the rarity of these incidents, one should consider white phosphorus as a differential diagnosis in case of self-igniting objects among both civilian and military burn victims. Awareness among military clinicians should be emphasized with advanced burn life support courses required to meet the unique needs of military personnel.
